# Children’s and Families’ Determinants of Health-Related Behaviors in an Italian Primary School Sample: The “Seven Days for My Health” Project

**DOI:** 10.3390/ijerph19010460

**Published:** 2022-01-01

**Authors:** Francesco Sanmarchi, Francesco Esposito, Sofia Marini, Alice Masini, Susan Scrimaglia, Angelo Capodici, Fabrizio Arrichiello, Filippo Ferretti, Marilisa Rangone, Francesca Celenza, Emilia Guberti, Domenico Tiso, Antonello Lorenzini, Laura Dallolio

**Affiliations:** 1 Specialty School of Hygiene and Preventive Medicine, University of Bologna, 40126 Bologna, Italy; francesco.sanmarchi@studio.unibo.it (F.S.); francesco.esposito32@studio.unibo.it (F.E.); susan.scrimaglia@studio.unibo.it (S.S.); angelo.capodici@studio.unibo.it (A.C.); 2 Department of Biomedical and Neuromotor Sciences, University of Bologna, 40126 Bologna, Italy; sofia.marini2@unibo.it (S.M.); fabrizio.arrichiello@studio.unibo.it (F.A.); marilisa.rangone@studio.unibo.it (M.R.); antonello.lorenzini@unibo.it (A.L.); laura.dallolio@unibo.it (L.D.); 3 Department of Public Health, Bologna Health Trust, 40126 Bologna, Italy; Filippo.ferretti@ausl.bologna.it (F.F.); francesca.celenza@ausl.bologna.it (F.C.); 4 Hygiene and Nutrition Service, Department of Public Health, 40126 Bologna, Italy; emilia.guberti@gmail.com; 5 Hospital Villa Maria, 47900 Rimini, Italy; dottortiso@gmail.com

**Keywords:** Mediterranean diet, physical activity, sedentary behavior, KIDMED index, children, lifestyle, parents, educational level

## Abstract

Childhood obesity is an established health problem, and there is a growing need for health promotion interventions focused on healthy behaviors in collaboration with parents and schools. The Mediterranean diet (MD) could help to tackle obesity, but it is essential to maintain a good level of physical activity (PA) and limit time spent in sedentary activities (ST). To explore family determinants, adherence to the MD and PA levels as potential predictors of a child’s health-related behaviors, we performed a cross-sectional analysis of 368 Italian primary school children with a mean age of 8.95 years (SD = 1.43). Data were collected from May to June 2017 using a weekly diary, an interactive tool to assess the child’s and parents’ lifestyle. The child’s degree of adherence to the MD was calculated using the KIDMED index. Adherence to the MD was high, medium and poor in 5.2%, 62.5% and 32.3% of children, respectively. Higher maternal educational level was positively associated with children’s MD and PA (*p* < 0.05) and negatively correlated to ST. Maternal fruit and vegetable consumption was positively related to the MD and negatively related to ST (*p* < 0.05). Maternal PA was positively associated with the MD (*p* < 0.001). Paternal PA, and fruit and vegetable consumption, were positively associated with children’s PA (*p* < 0.05). Our results underline the need for future studies, mainly focused on school-based and family-based interventions, to promote healthy lifestyles and nutritional habits.

## 1. Introduction

The prevalence of overweight and obesity in children and adolescents is an established health problem [1], particularly in high income countries, as defined by the World Bank [2]. Childhood obesity represents a major issue in these countries [3,4], where it has increased considerably over the last 30 years [5].

Suitable interventions are needed to fight this global obesity pandemic. Specifically, there is a need for a cultural shift in terms of food choice, and reconciliation of the idea of a modern lifestyle with a healthy one. Admittedly, these challenges cannot be addressed without assessing the importance of schools and their curricula, particularly for their pivotal role in educating children about healthy behaviors and lifestyles [6]. Alongside schools, parental food habits and feeding strategies are closely related to obesity in children [7,8,9]. This has been discussed by many authors who linked parents’ socioeconomic determinants with their children’s eating behaviors, food choices and body mass index (BMI) [7,8,9,10,11,12,13]. In this context, the Mediterranean dietary (MD) regimen [14], which is characterized by a high intake of fruits, vegetables, pulses, unprocessed cereals and extra-virgin olive oil, has been studied extensively as a tool to counter various risks of developing diseases (e.g., cardiovascular, endocrinological and psychiatric), and improve health status and academic performance [15,16]. This type of diet has been described as highly beneficial to health, and various tools have been developed to assess adherence to this food regimen in children and adolescents. Among these tools, one of the most relevant is the KIDMED questionnaire. Its index, based on the values assigned to administered questions, can be used to extract three levels of dietary adherence [17,18].

Alongside a healthy diet, it is essential to maintain a good level of physical activity (PA) and limit the time spent engaging in sedentary activities (e.g., watching TV, playing video games, etc.), to ensure a good state of health for children and adolescents. The World Health Organization (WHO) suggests replacing any sedentary behavior with physical activity, and to perform at least 60 min of moderate-to-vigorous PA (MVPA) every day, for children and adolescents from age 5 to 17 [19]. Plenty of literature shows that physical activity is strongly related to both health benefits [20,21] and positive psychosocial outcomes [22]; conversely, sedentary time is related to negative social, physical, and psychological health outcomes [23,24]. Moreover, parental influence plays a crucial role in children’s sedentary behaviors [25,26], whereas the association between parents’ and children’s PA, and the correlation between socioeconomic status (SES) and PA behaviors, are still debated.

In this scenario, the “Seven days for my health” project aimed to explore children’s and families’ determinants as potential predictors of health-related behaviors among primary school children living in the province of Bologna. The rationale behind this project is to develop a simple tool that can be used to improve family self-consciousness of their own nutritional habits and health conditions and, at the same time, promote healthy behavior in primary school. In particular, this study aims to evaluate the association between parental characteristics (SES, BMI, lifestyle, nutrition and physical activity) and children’s behaviors regarding diet and physical activity among a sample of school children living in Northern Italy.

## 2. Materials and Methods

### 2.1. Study Design and Participants

This cross-sectional study was conducted as part of the “Seven days for my health” project, in the primary school of Calderara di Reno, a small town in the province of Bologna in Emilia-Romagna, a Northern Italian Region with approximately 202,540 primary school children. The “Seven days for my health” project was endorsed by the University of Bologna (Italy), and the University of Bologna Bioethics Committee approved the study on 30 June 2016. The study was conducted following the Declaration of Helsinki.

Children from the 1st to the 5th grades were invited to participate in the project. No further inclusion/exclusion criteria were adopted. Informed consent from the parents and permission from the schools’ principals were required for children to participate in the study. This study follows the Strengthening the Reporting of Observational Studies in Epidemiology (STROBE) reporting guidelines [27].

### 2.2. Study Variables and Instruments

Data were collected from May to June 2017 using the diary “Seven days for my health” authored by Domenico Tiso. The aforementioned diary is an interactive tool used to assess children’s and parents’ lifestyles, including nutrient and food intake [28,29,30]. The diary covers the seven weekdays. Diaries were collected anonymously, evaluated for accuracy and completeness by researchers, and analyzed. Children completed the questionnaire under the supervision of a caregiver, which helped to reduce the risk of bias. The “Seven days for my health” diary includes five different sections:

Section 1—nationality and anthropometric characteristics of the child (age, gender, height and weight).

Section 2—child’s weekly physical activity and daily screen time.

Section 3 and Section 4—parents’ or legal guardians’ weight, height, education level, occupation, physical activity and dietary habits.

Section 5—children’s daily dietary habits (specifically, breakfast, mid-morning snack, lunch, mid-afternoon snack and dinner).

Before completing the questionnaire, standardized pictures were shown to both children and parents to clarify the definition of a portion.

The adherence to the Mediterranean diet was calculated using the Diet Quality Index for Children and Adolescents (KIDMED) score (0 to 12) [17]. Subjects were classified into three levels of adherence to the Mediterranean diet: (1) >8, high; (2) 4–7, medium and (3) ≤3, poor.

Children’s PA levels were categorized following WHO guidelines of 60 min per day of MVPA [19]. Following the same WHO guidelines, parents’ PA levels were categorized as physically active if they performed 150 min per week of moderate-to-vigorous PA.

BMI was calculated as weight (in kilograms) divided by the square of height (in meters). Anthropometric status, stratifying by age and sex, was calculated according to the International Obesity Task Force (IOTF) cut-off values [31,32].

### 2.3. Data Analysis

Continuous variables were described using mean and standard deviation (±SD), while categorical variables were described through absolute and relative frequencies. Normal distribution of dependent variables was assessed graphically using density graphs and tested with the Shapiro–Wilk test.

The outcome variables investigated in this study were the following:

− Child’s IOTF categories (normal weight, overweight/obese)
− Child’s weekly average KIDMED index (0 to 12)
− Child’s PA level (less than 60 min per day, 60 min or more per day)
− Child’s daily screen time (minutes)

The associations between continuous variables and the variables of interest were analysed using the Student *t*-test on means or the analysis of variance (ANOVA) when 3 or more groups were compared. Multiple linear regression models with backward stepwise selection were then employed to identify the independent factors associated with the continuous outcome variables. Results from the linear regression were reported as regression coefficients (b) and relative 95% confidence intervals (95% CIs).

The associations between dichotomous variables and the variables of interest were assessed with the Pearson’s Chi-square test, followed by the estimation of a multiple logistic regression model with backward stepwise selection. Results from the logistic regression were reported as odds ratio (OR) and 95% CI. Results from the univariate analysis can be found in the Supplementary Materials (Table S1).

Regression models were developed using a set of 13 predictors (variables of interest):

− Child’s gender (male, female)
− Child’s age at the time of the survey (years)
− Mother’s and father’s age at the time of the survey (years)
− Mother’s and father’s BMI class (normal weight vs. overweight/obese)
− Mother’s and father’s education level (middle school or lower, high school, university degree or higher)
− Mother’s employment status (employed, unemployed)
− Mother’s and father’s physical activity (less than 150 min per week (yes), 150 min per week or higher(no))
− Mother’s and father’s portions of fruit and vegetables per day (number of portions > 5)

Regression models always included the following covariates: child’s gender and age. The significance level was set as *p* < 0.05. Questionnaires with missing data on the dependent variables and questionnaires with >20% missing data on the other variables were excluded from the analysis. Variables with >25% missing data (calculated separately for each section) were excluded from the analysis. Due to the presence of missing covariate data, multiple imputation by chained equations was used to replace missing values with multiple sets of simulated values to complete the data [33].

All analyses were carried out using R version 4.1.0 (R Project for Statistical Computing; (R Foundation for Statistical Computing, Vienna, Austria) [34].

## 3. Results

Out of the 428 administered questionnaires, 25 were discarded because the outcome variables were not reported, and 35 were discarded since they had more than 20% missing data in the entire questionnaire. In total, 368 questionnaires were eligible for the analysis.

The study population consisted of 197 girls and 171 boys aged 6 to 10 years (8.95 ± 1.43). The majority of the population had normal weight (*n* = 298; 81%) and the remaining part had overweight/obesity (*n* = 70; 19%).

Detailed population characteristics are summarized in Table 1.

### 3.1. Lifestyle, Nutrition and Physical Activity

The average weekly KIDMED index computed for each participant was 4.37 (SD = 1.80). Among the 368 participants, 204 (55.4%) met the WHO recommendation on fruit and vegetable consumption (5 a day).

The average time spent physically active was 99.1 min per day (SD = 53.7).

On average, children had 87.2 (SD = 70.2) minutes of daily screen time, with 272 children spending less than 120 min in front of a screen (73.9%). In total, 225 (61.1%) children met the target of a healthy way of spending free time (more than 60 min of physical activity and less than 120 min of screen time).

### 3.2. Familial Predictors of Child’s IOTF Category

Logistic regression models were used to investigate how a child’s IOTF category (normal weight vs overweight/obese) is influenced by familial predictors.

The results show that both the mother’s (*p* = 0.034) and father’s (*p* = 0.021) BMI are positively related to a higher chance for the children of having an overweight/obesity condition (Table S2).

### 3.3. Maternal Predictors

Regression models were used to determine how maternal predictors affected the child’s health-related behaviors. Results are shown in Table 2. Higher maternal educational level (EL) (university degree or higher) is positively associated with both child’s weekly average KIDMED index (*p* = 0.020) and PA (*p* = 0.041), while it is negatively related to screen time (ST) (*p* = 0.013). Maternal fruit and vegetable consumption is positively related to the KIDMED index (*p* = 0.014) and negatively related to screen time (ST) (*p* = 0.008). Finally, maternal PA is positively associated with a higher weekly average KIDMED index (*p* < 0.001).

### 3.4. Paternal Predictors

The analysis performed with regression models (Table 2) showed that paternal fruit and vegetable consumption is positively associated with a child’s PA (*p* = 0.046). Moreover, paternal PA level (at least 150 min per week) is positively related to a higher child’s PA (*p* = 0.022).

### 3.5. Child’s Predictors

Regression models (Table 2) were used to determine how the child’s predictors affected the child’s health-related behaviors. Being female is significantly associated with lower ST (*p* = 0.035). A child’s older age is associated with a higher time spent watching tv, sitting at a pc or playing video games (*p* = 0.001). Albeit not significantly, older age is positively related to a higher chance of being physically active for 60 min per day or more (*p* = 0.057).

## 4. Discussion

The purpose of this cross-sectional study was to investigate the association between parents’ lifestyle determinants and children’s dietary habits and physical activity levels.

Out of the 368 children included in the study, 19.0% presented an overweight/obese condition. This finding differs from data reported by the Italian epidemiological surveillance systems, “OKkio alla SALUTE”, which showed that 29.8% of children of the Emilia-Romagna region had an overweight or obese condition in 2019 [35]. This could be explained by the higher physical activity levels and lower screen time recorded in our population in comparison to the ones reported in the 2019 “OKkio alla Salute”. In fact, 83.4% of the study population was engaged in physical activities for 60 min or more per day. Plenty of literature shows that, on average, the majority of children do not meet the recommendations for physical activity (60 min daily) or screen time (less than 120 min daily) [30,36,37,38], a stark contrast with our sample.

The fact that children reported both in-school activities, as well as out-of-school ones, could lead to different results. Considering the lower percentage of children with overweight/obesity in our population, we consider our results to be reliable, even though they are not in line with other studies. Moreover, 26.1% of children reported 2 or more hours of screen time per day, whereas the 2019 “OKkio alla SALUTE” release showed that 45% of their sample of Emilia-Romagna children reported 2 or more hours of screen time per day [35]. This finding can be explained by the study area’s environmental factors, specifically urban versus rural areas and leaf area index. Indeed, living in a rural area with a higher leaf area index can lead to lower screen time [39].

Adherence to the Mediterranean diet (MD), investigated using the KIDMED index, showed that more than half (67.8%) of our sample had a healthy and balanced diet. Our findings are aligned with Roccaldo and colleagues’ study performed on 1740 Italian children which reported that only 32% had poor adherence to the MD [40].

Plenty of literature shows that adherence to the Mediterranean diet is consistently related to demographic and social characteristics of the study participants and of both of their parents, without analysing them separately [41,42,43].

The results of our univariate analysis, as confirmed by previous evidence, show that a child’s behavior and lifestyle is predominantly related to the mother’s features and that the only two paternal factors associated with a child’s lifestyle were the father’s PA level and fruit or vegetable consumption [44,45,46]. The predominance of maternal influence on a child’s behavior could be related to the tendency of Italian mothers to spend more time than other family members with their children [47]. These findings show that it is crucial to increase awareness about the importance of the family environment, especially in mothers with a lower level of education.

Since a positive correlation between the father’s diet adherence and PA was found, our results seem to suggest that the father’s determinants influence aspects concerning their children’s spare time. Indeed, children whose fathers were adherent to the MD and physically active had a higher PA and a lower ST. A limited number of studies suggest that father’s PA may affect the children’s, although it is not always clear whether it is in a positive or a negative way [48]. This could be explained by a father’s greater involvement in this aspect of life compared to a mother’s, and that perhaps a more active father may encourage his child to be active. This is even supported by other researchers, who suggest that fathers can influence their children by being physically active through co-activity and direct observation of their own PA behavior [49]. Therefore, intervention to encourage fathers to practice PA with their children could increase children’s PA levels and reduce children’s inactivity.

Clearly, both parents influence the development of their children’s lifestyle choices, and each of them can influence in different ways, depending on their own characteristics [50]. Thus, it is important to involve both parents in lifestyle education through appropriate programs that consider the different parental elements that are more associated with a healthy lifestyle. In this scenario, schools may be a great setting to achieve this goal.

Besides the parents’ influence, there are some specific characteristics that need to be considered. Our analysis showed that older children had a higher ST compared to younger children and, in general, males had a higher ST than females. It is curious to note that children, while growing up, seem to be more interested in spending time in front of a screen; perhaps this is because it becomes easier for older children to obtain access to this type of device, especially when they are not at school. This could be responsible for increasing inactivity in older children; therefore, a target intervention in older children could be useful to decrease the time spent engaging in this type of activity at home.

It should be noted that our study evaluated children’s eating habits and physical activity during the entire week; therefore, it encompasses both in-school and out-of-school habits. Nowadays, schools still represent an ideal setting to promote healthy behaviors, since they gives access to children regardless of age, ethnicity, gender and socio-economic status. Furthermore, school can play an important role in shaping children’s healthy behaviors and in helping students to adopt an active lifestyle [51].

The development, implementation, and evaluation of effective and sustainable interventions that allow children to modify their lifestyles and adopt more healthy and active habits have become—in many countries—a key public health priority [52].

It is widely known that children’s habits are influenced by school education [51], as this environment helps to give valuable lessons in terms of education and lifestyle choices; however, parents also contribute to modifying their children’s habits. Nevertheless, we recognize that the investigated children’s habit indicators were linked to many parental variables. Thus, it would be wise to organize school-based educational meetings to raise parents’ awareness of the importance of promoting healthy behaviors.

Until today, the majority of school-based interventions which focused on health and wellbeing were centered on the school environment, despite the idea that multicomponent interventions involving the family or community, in addition to the school, are likely to be most effective. Family support can influence out-of-school physical activity [53], and the majority of the food that children consume originates from the home [54,55]. For these reasons, it is increasingly essential to consider the family environment in order to achieve long-term lifestyle changes. Indeed, parents, families, home environment and school have predominant roles in influencing children’s eating behaviors and physical activity. The study had several limitations that should be acknowledged. Firstly, this study is not representative of the whole Italian population. Secondly, the questionnaires were filled in from May to June and it is possible that, during the colder months of the year, children had different diet and lifestyle habits. Lastly, although the self-reported questionnaires were filled in by the children with the supervision of an adult, the risk of bias cannot be eliminated.

## 5. Conclusions

In conclusion, in our sample, children’s lifestyle is influenced by both parents; however, each parent affects the eating and physical habits of their child differently, according to their own characteristics. Therefore, parents are a vital target group to sensibilize, and lifestyle intervention should not be limited to children, but include both parents to achieve health improvement for the whole family

Our results underline the need for future studies mainly focused on school-based intervention and family-based intervention, targeted to the promotion of healthy lifestyles.

## Figures and Tables

**Table 1:** Population characteristics (N = 368).

Population Characteristics	N (%)
Child’s gender	
Male	171 (46.5%)
Female	197 (53.5%)
Age (years)	8.95 (1.43)
IOTF category	
Normal weight	298 (81.0%)
Overweight/Obesity	70 (19.0%)
Daily PA time, mean (SD)	99.1 (53.7)
More than 60 min of PA per day	
No	61 (16.6%)
Yes	307 (83.4%)
KIDMED index, mean (SD)	4.37 (1.80)
KIDMED index category	
Low (<4)	119 (32.3%)
Medium (4–6)	230 (62.5%)
High (>6)	19 (5.2%)
Daily screen time (minutes)	87.16 (70.25)
Daily screen time <120 min	272 (73.9%)
Daily screen time < 120 min and more than 60 min of PA per day	225 (61.1%)
Fruit & vegetables consumption per day (portions), mean (SD)	2.11 (0.88)
Meeting WHO recommendation on fruit and vegetable consumption	204 (55,4%)
Mother’s BMI category	
Normal weight	280 (76.1%)
Overweight/Obesity	88 (23.9%)
Father’s BMI category	
Normal weight	152 (41.3%)
Overweight/Obesity	216 (58.7%)
Mother’s educational level	
Middle school or lower	73 (19.8%)
High school	185 (50.3%)
University degree or higher	110 (29.9%)
Father’s educational level	
Middle school or lower	105 (28.5%)
High school	192 (52.2%)
University degree or higher	71 (19.3%)
Mother’s occupational status	
Unemployed	53 (14.4%)
Employed	315 (85.6%)
Father’s occupational status	
Unemployed	5 (1.4%)
Employed	363 (98.6%)
Mother’s PA per week (minutes)	
Less than 150	260 (70.7%)
150 or more	108 (29.3%)
Father’s PA per week (minutes)	
Less than 150	247 (67.1%)
150 or more	121 (32.9%)
Mother’s fruit & vegetable consumption per day (portions)	3.43 (1.23)
Father’s fruit & vegetable consumption per day (portions)	3.09 (1.28)

Body Mass Index (BMI), International Obesity Task Force (IOTF), Physical Activity (PA), Standard Deviation (SD).

**Table 2:** Regression models results.

		Child’s KIDMED Index			Child’s Screen Time			Child’s Physical Activity		
		Beta	95% CI	*p*	Beta	95% CI	*p*	OR	95% CI	*p*
Mother’s variables	Education									
	≤Lower secondary	-	-	-	-	-	-	-	-	-
	Higher secondary	0.116	−0.368, 0.601	0.637	−12.5	−31.1, 6.13	0.188	1.93	0.949, 3.92	0.069
	Degree or higher	0.633	0.103, 1.16	0.020	−29.7	−50.0, −9.41	0.004	2.83	1.824, 4.08	0.041
	Fruit & vegetable consumption	0.191	0.039, 0.342	0.014	−7.61	−13.6, −1.60	0.0013			
	PA per week (minutes)									
	Less than 150	-	-	-						
	150 or more	0.721	0.315, 1.13	<0.001						
Father’s variables	Fruit & vegetable consumption							1.27	1.176, 1.66	0.046
	PA per week (minutes)									
	Less than 150				-	-	-	-	-	-
	150 or more				−13.9	−29.4, 1.58	0.058	2.31	1.13, 4.72	0.022
Child’s variables	Gender									
	Male				-	-	-			
	Female				−15.1	−29.2, −1.09	0.035			
	Age				8.23	3.35, 13.1	0.001	1.22	0.994, 1.49	0.057

Confidence Interval (CI); Odds Ratio (OR); Physical Activity (PA).

## Data Availability

The data presented in this study are available on request from the corresponding author. The data are not publicly available due to ethical and privacy reasons.

## Supplementary Materials

The following supporting information can be downloaded at: https://www.mdpi.com/article/10.3390/ijerph19010460/s1. Table S1: Univariate analysis. Body Mass Index (BMI), International Obesity Task Force (IOTF), Physical Activity (PA); Table S2: Logistic regression model. Child’s BMI predicted by parental BMI. Body Mass Index (BMI), confidence interval (CI).
