# Supplementary material for: Children’s and Families’ Determinants of Health-Related Behaviors in an Italian Primary School Sample: The “Seven Days for My Health” Project
Source: Int J Environ Res Public Health. 2022 Jan 1;19(1):460. doi: 10.3390/ijerph19010460 (PMC8744665; doi:10.3390/ijerph19010460)
Supplement: Supplementary file 1 [file ijerph-19-00460-s001.zip › ijerph-1502928-supplementary.pdf]

Table S1. Univariate analysis. Body Mass Index (BMI), International Obesity Task Force (IOTF), Physical Activity (PA).

	Characteristics	N = 368[1]	Child's Kidmed index; n ± SD	p-value	Child's Screen Time; minutes ± SD	p-value	Child's Physical Activity > 60 min/day; n (%)	p-value
Mother's variables	**BMI category**			0.094		0.446		0.764
	Normalweight	280 (76%)	4.46±1.81		84.48±65.41		235 (84%)	
	Overweight/Obese	88 (24%)	4.10±1.74		95.68±83.68		72 (82%)	
	**Education**			0.007		0.044		0.225
	≤ Lower Secondary	73 (20%)	4.18±1.88		104.13±93.09		56 (77%)	
	Higher secondary	185 (50%)	4.22±1.80		89.18±68.70		157 (85%)	
	University Degree or higher	110 (30%)	4.76±1.69		72.49±50.07		94 (85%)	
	**Employment Status**			0.768		0.863		0.775
	Employed	315 (86%)	4.38±1.80		85.11±64.14		264 (84%)	
	Unemployed	53 (14%)	4.34±1.80		99.30±99.07		43 (81%)	
	**More than 150 minutes of PA per week**			<0.001		0.581		0.096
	Yes	108 (29%)	4.91±1.66		79.91±54.49		96 (89%)	
	No	260 (71%)	4.15±1.81		90.17±75.73		211 (81%)	
Father's Variables	**BMI category**			0.864		0.525		0.181
	Normalweight	152 (41%)	4.42±1.91		89.16±71.24		132 (87%)	
	Overweight/Obese	216 (59%)	4.34±1.72		85.75±69.67		175 (81%)	
	**Education**			0.677		0.989		0.829
	≤ Lower Secondary	105 (28%)	4.36±1.86		89.51±78.36		89 (85%)	
	Higher secondary	192 (52%)	4.35±1.85		87.87±71.62		158 (82%)	
	University Degree or higher	71 (20%)	4.44±1.59		81.73±52.08		60 (85%)	

	Characteristics	N = 368[1]	Child's Kidmed index; n ± SD	p-value	Child's Screen Time; minutes ± SD	p-value	Child's Physical Activity > 60 min/day; n (%)	p-value
Child's Variables	**More than 150 minutes of PA per week**			0.031		0.177		0.024
	Yes	121 (33%)	4.67±1.91		78.01±56.60		109 (90%)	
	No	247 (67%)	4.23±1.73		91.64±75.75		198 (80%)	
	**Sex**			0.266		0.017		>0.999
	Female	197 (54%)	4.44±1.70		80.37±67.93		164 (83%)	
	Male	171 (46%)	4.30±1.91		94.97±72.24		143 (84%)	

Table S2. Logistic regression model. Child's BMI predicted by parental BMI. Body Mass Index (BMI), confidence interval (CI).

	**Beta**	**95% CI[1]**	**p-value**
Mother's BMI	1.06	1.02, 1.14	0.034
Father's BMI	1.12	1.03, 1.21	0.021
